# Frequency and functional profile of circulating TCRαβ^+^ double negative T cells in HIV/TB co-infection

**DOI:** 10.1186/s12879-022-07807-3

**Published:** 2022-11-28

**Authors:** Yuting Tan, Shi Zou, Wei Guo, Yanni Xiang, Yu Dong, Qi Zhu, Songjie Wu, Mingqi Luo, Ling Shen, Ke Liang

**Affiliations:** 1 Department of Infectious Diseases, Zhongnan Hospital of Wuhan University, Wuhan, China; 2 Wuhan Research Center for Infectious Diseases and Cancer, Chinese Academy of Medical Sciences, Wuhan, China; 3 Department of Pathology, Zhongnan Hospital of Wuhan University, Wuhan, China; 4 Department of Pathology, School of Basic Medical Sciences, Wuhan University, Wuhan, China; 5 Department of Intensive Care Medicine, Yichang Central People’s Hospital, Yichang, Hubei China; 6 Department of Geriatrics, Zhongnan Hospital of Wuhan University, Wuhan, China; 7 Wuhan Pulmonary Hospital, Wuhan Institute for Tuberculosis Control, Wuhan, China; 8 Department of Nosocomial Infection Management, Zhongnan Hospital of Wuhan University, Wuhan, China; 9 Department of Microbiology and Immunology, Center for Primate Biomedical Research, University of Illinois College of Medicine, Chicago, IL USA; 10 Hubei Engineering Center for Infectious Disease Prevention, Control and Treatment, Wuhan, China

**Keywords:** Human immunodeficiency virus, Tuberculosis, TCRαβ^+^ double negative T cells, Fas, CCR5, Granzyme A

## Abstract

**Background:**

Increased frequency of circulating double negative T (DNT, CD4^−^CD8^−^CD3^+^) cells with protective immune function has been observed in human immunodeficiency virus (HIV) infection and tuberculosis (TB). Here the role of circulating TCRαβ^+^ DNT cells was further investigated in HIV/TB co-infection.

**Methods:**

A cross-sectional study was conducted to investigate the frequency and functional profiles of peripheral TCRαβ^+^ DNT cells including apoptosis, chemokine and cytokine expression among healthy individuals and patients with TB, HIV infection and HIV/TB co-infection by cell surface staining and intracellular cytokine staining combined with flow cytometry.

**Results:**

Significantly increased frequency of TCRαβ^+^ DNT cells was observed in HIV/TB co-infection than that in TB (p < 0.001), HIV infection (p = 0.039) and healthy controls (p < 0.001). Compared with TB, HIV/TB co-infection had higher frequency of Fas expression (p = 0.007) and lower frequency of Annexin V expression on TCRαβ^+^ DNT cells (p = 0.049), and the frequency of Annexin V expression on Fas^+^TCRαβ^+^ DNT cells had no significant difference. TCRαβ^+^ DNT cells expressed less CCR5 in HIV/TB co-infection than that in TB (p = 0.014), and more CXCR4 in HIV/TB co-infection than that in HIV infection (p = 0.043). Compared with healthy controls, TB and HIV/TB co-infection had higher frequency of TCRαβ^+^ DNT cells secreting Granzyme A (p = 0.046; p = 0.005). In TB and HIV/TB co-infection, TCRαβ^+^ DNT cells secreted more granzyme A (p = 0.002; p = 0.002) and perforin (p < 0.001; p = 0.017) than CD4^+^ T cells but similar to CD8^+^ T cells.

**Conclusions:**

Reduced apoptosis may take part in the mechanism of increased frequency of peripheral TCRαβ^+^ DNT cells in HIV/TB co-infection. TCRαβ^+^ DNT cells may play a cytotoxic T cells-like function in HIV/TB co-infection.

## Background

Tuberculosis (TB) caused by *Mycobacterium tuberculosis* (MTB) is one of the most common opportunistic infections and mortality of acquired immune deficiency syndrome (AIDS). Human immunodeficiency virus (HIV) and TB co-infection influence each other to promote disease progression. HIV infection increases the susceptibility to MTB and the risk of progression to active TB, in turn, TB promotes HIV replication and viral diversity, exacerbating AIDS progression [1].

Double negative T cells (DNT cells), defined as CD3^+^ T cells that lack of CD4 and CD8 expression, derive either from the thymus by the escape of negative selection or from CD4 + T cells or CD8 + T cells in the periphery in response to antigenic stimulation [2–4]. According to different T cell receptor (TCR), DNT cells can be divided into TCRαβ^+^ DNT cells and TCRγδ^+^ DNT cells [5, 6]. In humans and mice, about 95% of T cells express TCRαβ, and only a few (5%) express TCRγδ [6]. DNT cells only account for a low frequency (1%-5%) of peripheral T cells in general population, while increased frequency of peripheral DNT cells have been observed in autoimmune diseases, neoplastic diseases and infectious diseases [4, 7–9].

Previous study found in AIDS patients, the frequency of DNT cells in periphery increased significantly, which is twice that of healthy individuals [7]. Both peripheral and pulmonary mucosal DNT cells were reported to be the latent HIV virus reservoirs [10], and TCRαβ^+^ DNT cell was the major cell type harboring productive infection in peripheral blood [11]. However, whether HIV enters and infects DNT cells via HIV co-receptors is still unclear. Moreover, DNT cells were also associated with the control of immune activation and disease progression in HIV and simian immunodeficiency virus (SIV) infection [12, 13]. Elevated frequency of peripheral TCRαβ^+^ DNT cells were also observed in patients with TB compared with healthy controls, with a protective immunity function by secreting more IFN-γ [14]. In vivo study found pulmonary TCRαβ^+^ DNT cells could reduce the growth of MTB in the lungs [15].

Currently, the frequency and functional profile of peripheral TCRαβ^+^ DNT cells in HIV/TB co-infection is still unknown. Based on the role of TCRαβ^+^ DNT cells in HIV and TB pathogenesis and their ability to harbor persistent HIV reservoirs, we aim to investigate the frequency and functional profiles of peripheral TCRαβ^+^ DNT cells including apoptosis, chemokine and cytokine expression among patients with HIV/TB co-infection. Our preliminary study data may enrich and expend the role of DNT cells.

## Methods

### Study population

Adults (≥ 18 years old) who met the following inclusion criteria of HC, TB, HIV and HT group and volunteered to participate in this study were enrolled sequentially between May, 2018 and December, 2020. Individuals enrolled in this study were divided into four groups: (1) Healthy control (HC) group: healthy people without any history of chronic inflammatory diseases and infection manifestations within 2 weeks prior to enrollment were recruited from the physical examination center in Zhongnan Hospital of Wuhan University. HIV infection was excluded by HIV antibody screening and MTB infection was excluded by chest X-ray and Interferon Gamma Release Assay (IGRA). (2) Tuberculosis (TB) group: patients with confirmed TB by etiological or histopathological methods (smear or culture positive, and/or MTB DNA test positive, and/or Xpert MTB/RIF test positive, and/or histopathological evidence supporting TB) were recruited from Wuhan Pulmonary Hospital. All patients had been excluded HIV infection and hadn’t received anti-TB therapy prior to enrollment. (3) HIV infection (HIV) group: patients with confirmed HIV infection and without MTB infection by chest X-ray and IGRA screening were recruited from AIDS Clinical Guidance and Training Center, Zhongnan Hospital of Wuhan University All patients hadn’t received antiretroviral therapy (ART) prior to enrollment. (4) HIV/TB co-infection (HT) group: patients with HIV infection and active TB were recruited from department of infectious diseases, Zhongnan Hospital of Wuhan University. All patients hadn’t received ART and anti-TB therapy prior to enrollment.

### Isolation of PBMCs

EDTA coagulated peripheral blood were collected from all enrolled individuals and then peripheral blood mononuclear cells (PBMCs) were isolated with the Ficoll-Paque method. Cell pellets were treated with 5 ml RBC lysis buffer (Sigma-Aldrich) for 10 min followed by washing once with 5% FBS-PBS. PBMCs were counted and cryopreserved with fetal calf serum (FCS) containing 10% dimethyl sulfoxide (DMSO) at − 80 °C.

### Cell surface molecular staining

After resuscitation and counting, 1 × 10^6^ PBMCs were suspended with a 200μL final volume. For surface marker staining, cells were stained with specific antibodies for 30 min at 4 °C in the dark. The following antibodies were used: anti-CD3-PerCP-cy5.5 (clone UCHT1; Biolegend), anti-CD8-APC-Cy7 (clone SK1; Biolegend), anti-CD4-PE-Cy7 (clone RPA-T4; Biolegend), anti-TCRγδ-FITC (clone B1; Biolegend), anti-Fas-APC (clone DX2; BD Biosciences), Annexin V-PE (Annexin V PE Apoptosis kit, Cat#559763, BD Biosciences), anti-CCR5-PE (clone J418F1; Biolegend), anti-CXCR3-APC (clone G025H7; Biolegend), anti-CXCR4-PE (clone 12g5; Biolegend), anti-CCR7-APC (clone G043h7; Biolegend). The stained cells were fixed with 2% paraformaldehyde and then analyzed by flow cytometry.

### Direct intracellular cytokine staining

This procedure was performed as described before [16]. PBMCs were incubated for one hour stimulated with anti-CD28/CD49d (1 mg/ml) in a 200μL final volume medium in round-bottom 96-well plates at 37 °C and 5% CO_2_. Incubation with Brefeldin A (GolgiPlug, BD Biosciences) for 5 h was performed for further intracellular cytokines staining. Use PBS to wash cells twice and then surface marker staining was performed with with specific antibodies for 30 min at 4 °C in the dark. Fixation and Permeabilization Solution (Cytofix/Cytoperm; BD Biosciences) were added for 45 min at 4 °C in the dark. For intracellular cytokines staining, the following antibodies were used: anti-Granzyme A-PE (clone CB9; Biolegend), anti-Perforin-APC (clone dG9; Biolegend), anti-IFN-γ-PE (clone 4S.B3; Biolegend) and anti-TNF-α-APC (clone MAb11; Biolegend) according to the manufacturer's instructions. The stained cells were fixed with 2% paraformaldehyde and then analyzed by flow cytometry.

### Statistical analysis

SPSS 21.0 and Graphpad Prism 5.0 were used for data statistics and plotting. Variables are denoted as the median (range) or n (%). Non-parametric rank sum test was used for comparison between groups. p < 0.05 was considered statistically significant.

## Results

### Participants’ characteristics

A total of 218 individuals (49 in the HC group, 75 in the TB group, 45 in the HIV group and 49 in the HT group) were enrolled in this study. The basic characteristics of participants were shown in Table 1. The proportion of male in TB, HIV and HT group were higher than that in HC group, but had no significant difference between TB, HIV and HT group. Both HIV group and HT group had lower CD4^+^ T lymphocyte count (CD4 count) than HC group and TB group, while the CD4 count in HT group was the lowest among the four groups. The proportion of pulmonary TB and extrapulmonary TB between TB and HT group had no significant difference.

**Table 1:** Characteristics of the study participants

	HC group (n = 49)	TB group (n = 75)	HIV group (n = 45)	HT group (n = 49)	p value
Age [years, median (range)]	29 (24–50)	43 (25–56)	38 (27–47)	39 (31–49)	0.110
Male, n (%)	11 (31)	46 (77)	26 (87)	34 (85)	< 0.001
CD4^+^ T cell counts [/ul, median (range)]	898 (746–969)	743 (612–924)	400 (311–582)	88 (49–176)	< 0.001
Pulmonary TB, n(%)	/	57 (76)	/	30 (61)	0.060

*HC* healthy control, *TB* tuberculosis, *HT* HIV/TB co-infection

### TCRαβ^+^ DNT cells frequency in patients with HIV/TB co-infection

**Fig. 1:**
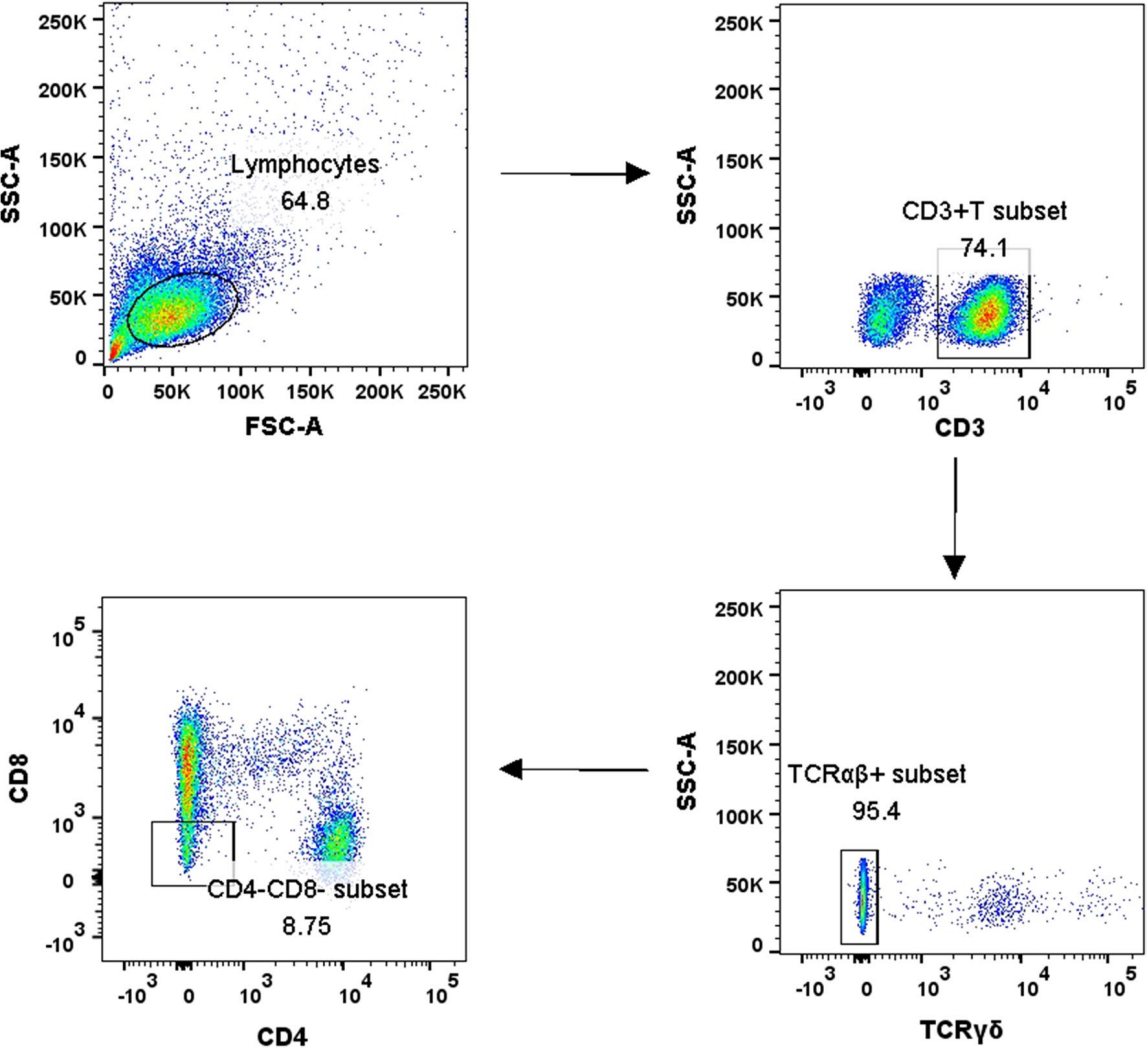
The flow cytometric gating strategy displayed in healthy individuals, patients with TB, patients with HIV infection and patients with HIV/TB co-infection

**Fig. 2:**
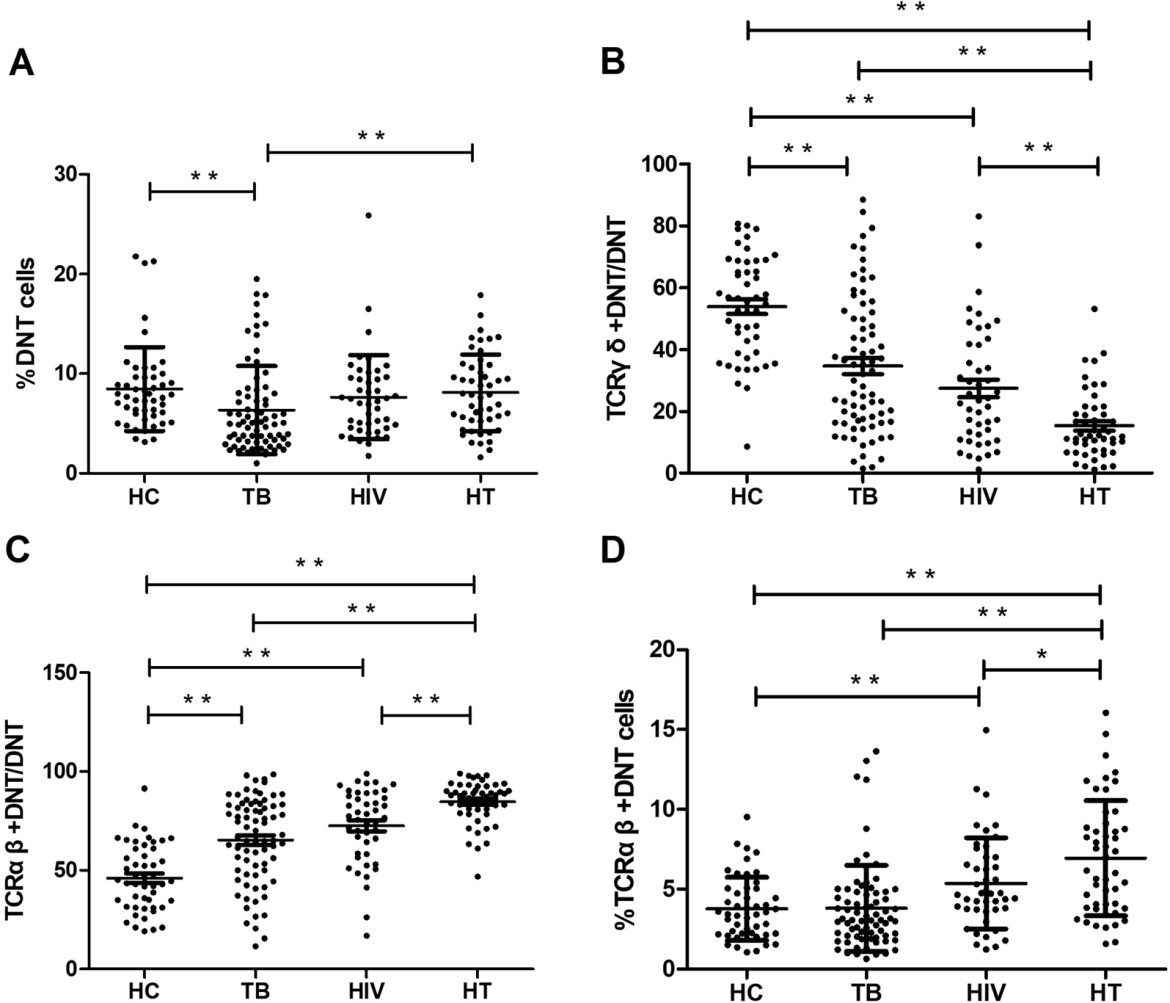
**A** Comparison of the DNT cells proportion to CD3^+^ T cells in HC, TB, HIV and HT group. **B** Comparison of the TCRγδ^+^ DNT cells proportion to DNT cells in HC, TB, HIV and HT group. **C** Comparison of the TCRαβ^+^ DNT cells proportion to DNT cells in HC, TB, HIV and HT group. **D** Comparison of the TCRαβ^+^ DNT cells proportion to CD3^+^ T cells in HC, TB, HIV and HT group. *p < 0.05; **p < 0.01. *HC* healthy controls, *TB* patients with tuberculosis, *HIV* patients with HIV infection, *HT* patients with HIV/TB co-infection

The flow cytometric gating strategy was displayed in Fig. 1. As shown in Fig. 2A, the frequency of DNT cells to CD3^+^ T cells in TB group was significantly lower than that in HC group (p < 0.001), and the frequency of DNT cells in HT group was significantly higher than that in TB group (p = 0.002). No significant difference of DNT cells frequency was found between HIV group and HT group.

Further subset analysis according to the TCR expression showed that the proportion of TCRγδ^+^ DNT cells to DNT cells was significantly decreased in HT group in comparison to HC, TB and HIV group (p < 0.001; p < 0.001; p = 0.001), only accounting for 12%; while the proportion of TCRαβ^+^ DNT cells to DNT cells was the highest in HT group in comparison to HC, TB and HIV group (p < 0.001; p < 0.001; p = 0.001), accounting for 88% (shown in Fig. 2B, C). These data suggested the expansion of TCRαβ^+^ DNT cells was the main contributor to the increased frequency of DNT cells in HT group, so we focused on analyzing the frequency and functional profile of peripheral TCRαβ^+^ DNT cells in our study. As shown in Fig. 2D, the frequency of TCRαβ^+^ DNT cells to CD3^+^T cells in HT group was significantly higher than that in TB group (p < 0.001), HIV group (p = 0.039) and HC group (p < 0.001).

### Fas expression on TCRαβ^+^ DNT cells in HIV/TB co-infection

**Fig. 3:**
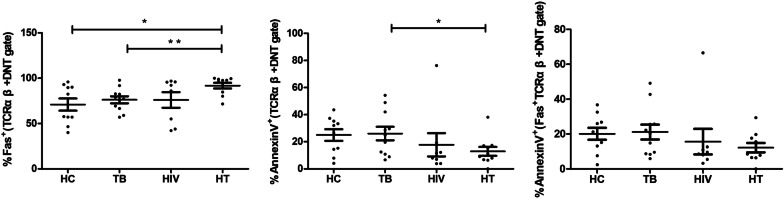
Comparison of TCRαβ^+^ DNT cells expressing Annexin V and Fas in HC, TB, HIV and HT group. *p < 0.05; **p < 0.01. *HC* healthy controls, *TB* patients with tuberculosis, *HIV* patients with HIV infection, *HT* patients with HIV/TB co-infection

The Annexin V and Fas expression of TCRαβ^+^ DNT cells were preliminarily analyzed to investigate whether increased TCRαβ^+^ DNT cells frequency was related to Fas mediated apoptosis. As shown in Fig. 3, the frequency of TCRαβ^+^ DNT cells with Annexin V expression in HT group was lower than that in TB group (p = 0.049). However, compared with the TB group (p = 0.007) and HC group (p = 0.010), HT group had higher frequency of TCRαβ^+^ DNT cells expressing Fas, and the frequency of Annexin V expression on Fas^+^TCRαβ^+^ DNT cells between HT group and TB group had no significant difference.

### CCR5 and CXCR4 expression on TCRαβ^+^ DNT cells in HIV/TB co-infection

**Fig. 4:**
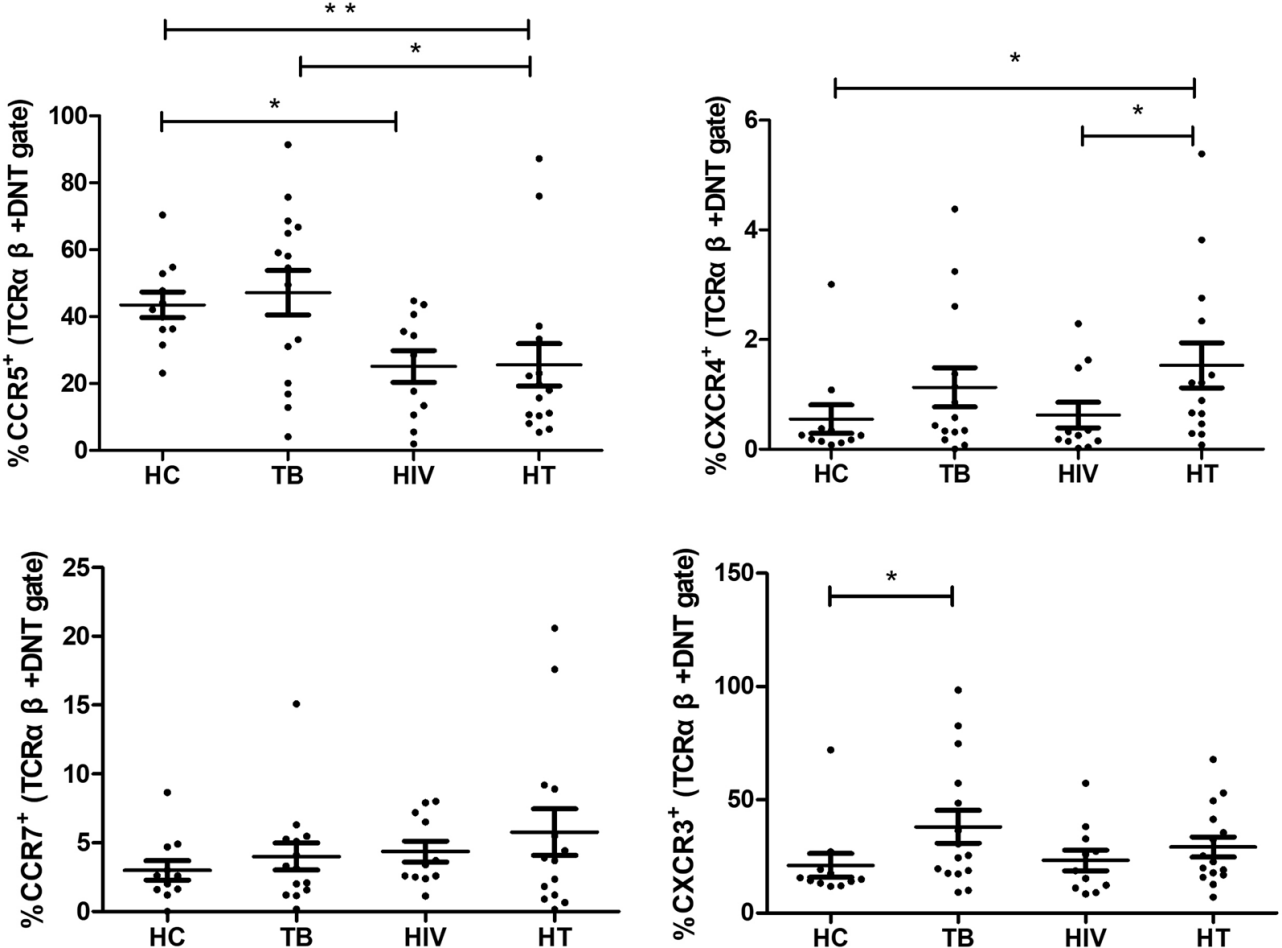
Comparison of TCRαβ^+^ DNT cells expressing CCR5, CXCR4, CCR7, CXCR3 in HC, TB, HIV and HT group. *p < 0.05; **p < 0.01. *HC* healthy controls, *TB* patients with tuberculosis, *HIV* patients with HIV infection, *HT* patients with HIV/TB co-infection

The frequencies of TCRαβ^+^ DNT cells expressing CC chemokine receptor and CXC chemokine receptor in each group were evaluated. As shown in Fig. 4, lower frequency of CCR5 expression on TCRαβ^+^ DNT cells was observed in HIV group (p = 0.014) and HT group (p = 0.005) than that in HC group, and lower frequency of CCR5 expression on TCRαβ^+^ DNT cells was observed in HT group than that in TB group (p = 0.036). Compared with HIV group (p = 0.043) and HC group (p = 0.013), TCRαβ^+^ DNT cells expressed more CXCR4 in HT group. No significant difference of TCRαβ^+^ DNT cells expressing CCR7 and CXCR3 were found between TB group, HIV group and HT group.

### Cytokines secretion of TCRαβ^+^ DNT cells in HIV/TB co-infection

**Fig. 5:**
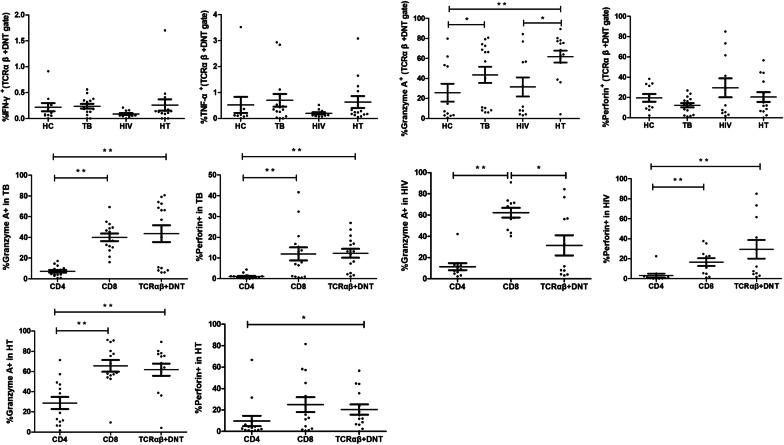
**A** Comparison of TCRαβ^+^ DNT cells secreting IFN-γ, TNF-α, granzyme A, perforin in HC, TB, HIV and HT group. **B** Comparison of CD4^+^ T, CD8^+^ T and TCRαβ^+^ DNT cells secreting granzyme A, perforin in TB, HIV and HT group. *p < 0.05; **p < 0.01. *HC* healthy controls, *TB* patients with tuberculosis, *HIV* patients with HIV infection, *HT* patients with HIV/TB co-infection

The function of cytokines secretion of TCRαβ^+^ DNT cells in each group were further analyzed. As shown in Fig. 5A, the frequency of TCRαβ^+^ DNT cells expressing granzyme A was significantly higher in HT group than that in HIV group (p = 0.025) and HC group (p = 0.005). TCRαβ^+^ DNT cells expressed more granzyme A in TB group than that in HC group (p = 0.046). No significant difference of TCRαβ^+^ DNT cells secreting perforin, IFN-γ and TNF-α were found between HC group, TB group, HIV group and HT group.

We further compared the cytokine secretion functions of CD4^+^ T cells, CD8^+^ T cells and TCRαβ^+^ DNT cells in TB group, HIV group and HT group, respectively. As shown in Fig. 5B, in TB group and HT group, TCRαβ^+^ DNT cells secreted more granzyme A (p = 0.002; p = 0.002) and perforin (p < 0.001; p = 0.017) than CD4^+^ T cells and the secretion of granzyme A and perforin between TCRαβ^+^ DNT cells and CD8 + T cells had no significant difference. In HIV group, TCRαβ^+^ DNT cells secreted more perforin than CD4^+^ T cells (p = 0.001) and less granzyme A than CD8^+^ T cells (p = 0.028). No significant differences of IFN-γ and TNF-α secretion between CD4^+^ T cells, CD8^+^ T cells and TCRαβ^+^ DNT cells were found in TB group, HIV group and HT group (Results not shown).

## Discussion

To our knowledge, this is the first study to investigate the frequency and function characteristics of peripheral TCRαβ^+^ DNT cells, especially the Fas, CCR5, CXCR4 expression on peripheral TCRαβ^+^ DNT cells in HIV infection with or without TB. High frequencies of DNT cells in HIV infection and TB has been described previously [12, 14]. In our study. we found that HIV/TB co-infection could lead to an expansion of peripheral TCRαβ^+^ DNT cells compared with mono-TB and mono-HIV infection. In HIV infected patients, TCRαβ^+^ DNT cells were thought to be derived both normally from within the thymus, and also from cells originally expressing CD4, but which have undergone CD4 downregulation in response to HIV-1 infection through the cooperative action of Env, Nef, and Vpu [17–19]. Hence, co-infection with HIV may drive the CD4 internalization of HIV infected CD4^+^ T cells and then increase the generation of TCRαβ^+^ DNT cells in patients with TB. Moreover, CD4 down-regulation might change the cell signaling pathways and thus prevent CD4^+^ T cells from HIV gp120 induced apoptosis [20, 21]. However, whether the expansion of TCRαβ^+^ DNT cells in HIV/TB co-infection is related to the decreased apoptosis remains unclear.

In our study, we found the apoptosis of TCRαβ^+^ DNT cells in HIV/TB co-infection was lower than that in TB according to the AnnexinV expression. Fas and Fas Ligand (FasL) pathway is one of the key death receptor signaling pathways involved in the regulation of apoptotic cell death [22, 23]. Fas and FasL pathway also contributed to the T cells apoptosis in HIV infection [24, 25]. It's worth noting that our study found the frequency of TCRαβ^+^ DNT cells expressing Fas was higher in HIV/TB co-infection than that in TB, and the AnnexinV expression on Fas positive TCRαβ^+^ DNT cells had no significant difference between HIV/TB co-infection and TB. All these data suggested that the increased Fas expression didn’t mediate the higher apoptosis of TCRαβ^+^ DNT cells in HIV/TB co-infection. Previous studies found HIV Nef could interact with apoptosis signal-regulating kinase 1 (ASK1) and then inhibit Fas-mediated apoptosis and protect HIV-infected CD4^+^ T cells from CD8^+^ T cells killing [26, 27]. Given that TCRαβ^+^ DNT cells could be derived from CD4^+^ T cells with CD4 expression downregulated by HIV Nef [17], HIV Nef maybe also contribute to the immune evasion of TCRαβ^+^ DNT cells by inhibiting Fas-mediated pro-apoptotic signaling. This may be an immune evasion strategy of HIV that facilitates the survival of TCRαβ^+^ DNT cells (latent reservoir cells) and the persistence of intracellular viral replication.

The HIV co-receptors CXCR4 or CCR5, are well-known for their interaction with the HIV-1 gp120 to promote HIV entering into the target cells [28, 29]. Our data found that the expression of CCR5 on TCRαβ^+^ DNT cells was lower in HIV/TB co-infection and HIV infection than that in healthy controls, and the expression of CCR5 on TCRαβ^+^ DNT cells was lower in HIV/TB co-infection than that in TB. All these results suggested HIV infection might downregulate the CCR5 expression on TCRαβ^+^ DNT cells. In fact, previous studies found except surface CD4 expression downregulated by HIV Nef, the surface CCR5 on HIV infected CD4^+^ T cells could also be reduced by HIV Nef via distinct cellular mechanisms [30, 31]. Downregulation of CCR5 on TCRαβ^+^ DNT cells might be a viral strategy to avoid superinfection by HIV and thus protect them from premature death. More importantly, the binding of HIV gp120 to CCR5 could activate Fas/FasL pathway, thereby increase the susceptibility of T cells to Fas-mediated apoptosis [32–34]. Hence, in our study CCR5 downregulation on TCRαβ^+^ DNT cells may reduce the susceptibility to Fas-mediated apoptosis and thus inhibit TCRαβ^+^ DNT cells death in HIV infection and HIV/TB co-infection. Previous study showed high CXCR4 expression was helpful for X4 HIV entering into the target cells [35]. As AIDS progresses, there is a shift in viral coreceptor use from CCR5 to CXCR4 [36]. Our study found co-infected with TB increased the CXCR4 expression on TCRαβ^+^ DNT cells compared with mono-HIV infection, suggesting that HIV/TB co-infection might promote the entry of HIV into TCRαβ^+^ DNT cells by CXCR4 but not by CCR5.

In our study, TCRαβ^+^ DNT cells secreting more granzyme A in HIV/TB co-infection than that in HIV infection and healthy controls, and TCRαβ^+^ DNT cells secreting more granzyme A in TB than that in healthy controls, suggesting that TCRαβ^+^ DNT cells might exhibit cytotoxic T cells-like function in TB no matter with or without HIV infection. Consistent with this, the analysis and comparison of granzyme A and perforin secretion of CD4^+^ T cells, CD8^+^ T cells and TCRαβ^+^ DNT cells also showed TCRαβ^+^ DNT cells might display cytotoxic T cells-like function in TB and HIV/TB co-infection. Although our study found TCRαβ^+^ DNT cells might effect as CD8^+^ T cells, it is still unknown whether TCRαβ^+^ DNT cells derive from CD8^+^ T cells in HIV/TB co-infection. In autoimmune diseases, it was found TCRαβ^+^ DNT cells might arise from CD8^+^ T cells and displayed a distinct cytokine production profile by producing proinflammatory mediators that include IL-1β, IL-17, IFN-γ, CXCL3, and CXCL2. Upon activation, the CD8 downregulation on CD8 + T cells not only changed the phenotype of CD8^+^ T cells, but also granted them inflammatory capacity [37].

## Conclusion

Our study showed that the apoptosis of TCRαβ^+^ DNT cells was not matched with their Fas expression. Whether it’s a strategy for viral immune evasion by inhibiting Fas-mediated pro-apoptotic signaling or reducing the susceptibility to Fas-mediated apoptosis and thus contribute to the persistent virus replication in TCRαβ^+^ DNT cells need further exploration. Reduced apoptosis may take part in the mechanism of increased frequency of peripheral TCRαβ^+^ DNT cells in HIV/TB co-infection. TCRαβ^+^ DNT cells may play a cytotoxic T cells-like function in HIV/TB co-infection.

## Data Availability

All data generated or analyzed during this study are included in this published article.

## Abbreviations

DNT: Double negative T
HIV: Human immunodeficiency virus
TB: Tuberculosis
MTB: *Mycobacterium tuberculosis*
AIDS: Acquired immune deficiency syndrome
TCR: T cell receptor
SIV: Simian immunodeficiency virus
HC: Healthy control
IGRA: Interferon gamma release assay
ART: Antiretroviral therapy
PBMCs: Peripheral blood mononuclear cells
FCS: Fetal calf serum
ASK1: Apoptosis signal-regulating kinase 1
